# Achieving synchronization with active hybrid materials: Coupling self-oscillating gels and piezoelectric films

**DOI:** 10.1038/srep11577

**Published:** 2015-06-24

**Authors:** Victor V. Yashin, Steven P. Levitan, Anna C. Balazs

**Affiliations:** 1 Department of Chemical Engineering, University of Pittsburgh, Pittsburgh, PA 15261, USA; 2 Department of Electrical and Computer Engineering, University of Pittsburgh, Pittsburgh, PA 15261, USA

## Abstract

Lightweight, deformable materials that can sense and respond to human touch and motion can be the basis of future wearable computers, where the material itself will be capable of performing computations. To facilitate the creation of “materials that compute”, we draw from two emerging modalities for computation: chemical computing, which relies on reaction-diffusion mechanisms to perform operations, and oscillatory computing, which performs pattern recognition through synchronization of coupled oscillators. Chemical computing systems, however, suffer from the fact that the reacting species are coupled only locally; the coupling is limited by diffusion as the chemical waves propagate throughout the system. Additionally, oscillatory computing systems have not utilized a potentially wearable material. To address both these limitations, we develop the first model for coupling self-oscillating polymer gels to a piezoelectric (PZ) micro-electro-mechanical system (MEMS). The resulting transduction between chemo-mechanical and electrical energy creates signals that can be propagated quickly over long distances and thus, permits remote, non-diffusively coupled oscillators to communicate and synchronize. Moreover, the oscillators can be organized into arbitrary topologies because the electrical connections lift the limitations of diffusive coupling. Using our model, we predict the synchronization behavior that can be used for computational tasks, ultimately enabling “materials that compute”.

We focus on polymer gels undergoing the oscillatory Belousov-Zhabotinsky (BZ) reaction^1^,^2^. Fueled by the internalized BZ reaction, the gels oscillate autonomously in size, resembling a beating heart. With multiple BZ gels, the coherence between these oscillations could be used to perform spatio-temporal processing and recognition tasks^3^,^4^,^5^. Furthermore, BZ gels are pressure-sensitive^6^,^7^, and thus, could be used as materials that respond to human actions. We envision this BZ-PZ material to have a cellular structure, where each cell contains a swollen gel-piezoelectric unit. Figure 1a shows two electrically connected gel-piezoelectric units, illustrating the simplest coupling that permits communication between the oscillators. The expansion of the oscillating BZ gel on the left deflects the piezoelectric cantilever, which produces an electrical voltage. The generated voltage in turn causes a deflection of the cantilever on the right; this deflection imposes a force on the underlying BZ gel that modifies its oscillations. In this manner, the oscillations of the two gels can eventually become synchronized.

It is important to note that the coupling among the hybrid BZ-PZ units does not require external energy input because the BZ gels’ volumetric oscillations and thus, the PZ cantilever bending, are driven by the chemical energy of the BZ reaction; furthermore, this system operates without any amplification or computer mediation. This is in contrast to the pulse-coupled chemical reactors^5^, which interact through computerized electric pumps and valves, with a system of light sensitive BZ beads that are organized into a virtual network by a computer algorithm^8^, or the electrically coupled continuously stirred tank reactors^9^,^10^ that require signal amplification. The fact that these small-scale BZ-PZ units operate without requiring external energy or control make these systems suitable for integration into an actual materials “fabric”.

The gel in the basic unit (Fig. 1b) is sufficiently small that it exhibits spatially uniform chemo-mechanical oscillations. The relatively rigid piezoelectric cantilever is sufficiently thin that it is deflected by the expanding gel. The cantilever’s parallel bimorph structure consists of two identical layers of a polarized piezoelectric material (Fig. 1c,d). The internal and external surface electrodes are connected in parallel (Fig. 1d).

To capture the dynamics of this basic unit, we couple equations for the volumetric oscillations of the BZ gel and bending elasticity of the piezoelectric bimorph beam. We assume that the gel is formed from poly(N-isopropylacrylamide) (PNIPAAm) chains containing grafted ruthenium metal-ion catalysts^1^, and describe the kinetics of the BZ reaction in terms of the concentrations of activator, *u*, oxidized catalyst, *v*, and volume fraction of polymer, *ϕ*. In small gel samples, diffusion of the dissolved activator throughout the gel occurs faster than variations of *u* and *v* due to the BZ reaction, and hence, the reaction kinetics is given by^11^:


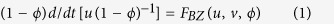



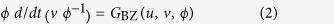


The functions *F*_BZ_ and *G*_BZ_ depend on the concentrations of *u* and *v*, and *ϕ* (see Supplementary Information). Periodic variations in *v* due to the BZ reaction affect the polymer-solvent interactions and drive the gel’s rhythmic expansion and contraction. The small gels equilibrate in size essentially instantaneously; the sample’s dimensions are determined by a balance among the elasticity of the network, force exerted by the cantilever, and osmotic pressure of the polymer^11^,^12^. For simplicity, we assume that the gel exhibits uniaxial deformations, which could be achieved by confining the gel within a capillary, and the force balance equation (Fig. 1e,f, and SI) becomes:


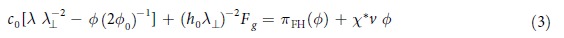


The first and second terms on the left-hand-side of eq. (3) are the elastic stress within the gel and the pressure exerted by the cantilever, respectively. The elastic stress is proportional to the gel crosslink density, *c*_0_, and depends on the gel’s degrees of swelling in the longitudinal, *λ*, and transverse, *λ*_⊥_, directions. The volume fraction of polymer is calculated as 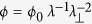, where *ϕ*_0_ is the polymer volume fraction in the un-deformed gel. Note that *λ*_⊥_ = *const* for the uniaxial deformation. The right-hand-side of eq. (3) gives the contributions to the osmotic pressure of the polymer according to the Flory-Huggins theory, *π*_FH_, and due to the hydrating effect of the oxidized catalyst that is controlled by the interaction parameter χ^*^ (see SI).

It is worth noting that in the previous studies^13^,^14^, we modeled the synchronization among pieces of BZ gels that were embedded in a chemically inert polymer network. These isolated BZ “patches” interacted with each other *via* the diffusion of *u* through the inert matrix, leading to a synchronization of the chemo-mechanical oscillations displayed by the BZ patches. The complementary experimental studies^14^,^15^ confirmed the predictions that emerged from our studies on the modes of synchronization in these heterogeneous systems.

The behavior of the piezoelectric bimorph driven by the BZ gel can be described by quasi-static equations because the frequency of the chemo-mechanical oscillations is much smaller than the eigenfrequency of the cantilever. These equations^16^ provide the deflection, *ξ*, and the electric charge, *Q*, of the bimorph as linear functions of the force, *F*, applied to the cantilever and the electric potential difference (voltage), *U*, between the electrodes (Fig. 1a):


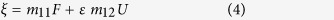



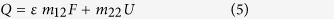


The force is applied to the cantilever’s tip in the vertical direction. The coefficients *m*_11_, *m*_12_, and *m*_22_ depend on the cantilever dimensions (Fig. 1c), and the properties of the piezoelectric material: the Young’s modulus *E*, piezoelectric constant *d*_31_, and dielectric constant *ε*_33_ (see SI). The force polarity, *ε*, on the r.h.s. of eqs. (4) and (5) is either +1 if the direction of polarization of the piezoelectric material (Fig. 1d) coincides with that of the applied force, or −1 if the polarization and force are in opposite directions. If the electrodes are shorted (*U* = 0), eq. (4) gives Hooke’s law, 
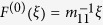
. In the open circuit configuration, where *Q* = 0, the bending rigidity of the cantilever is affected by the piezoelectric effect, so that *F*(*ξ*) = (1−*ζ*^2^)^−1^*F*^(0)^(*ξ*), where 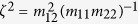. The value of *ζ* does not depend on the bimorph dimensions; namely, *ζ* = 3*k*(16−4*k*^2^)^−1/2^, where 
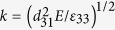
 is the electromechanical coupling factor characterizing the piezoelectric material (see SI). The effect of piezoelectricity on bending rigidity is small as *ζ*^2^ << 1 for a typical piezoelectric. We assume that the cantilever is fabricated from polarized Lead-Zirconate-Titanate ceramics (PZT), one of the most commonly used piezoelectrics^17^. Typically, *ζ*^2^ ≈ 0.04 for PZT; alternative processing methods^18^,^19^ can yield a two-fold increase in *d*_31_, and hence, a four-fold increase in *ζ*^2^.

The piezoelectric cantilever affects the chemo-mechanical oscillations in the BZ gel through the force *F*_*g*_ applied on the gel surface. Once the bending force *F*(*ξ*) is determined through eqs. (4) and (5), *F*_*g*_ as a function of *λ* is known, since *F*_*g*_ = *F*(*ξ*), and the cantilever deflection is caused by the gel swelling, so *ξ* = (*λ*−λ^*^)*h*_0_ (Fig. 1b). Hence, the dynamics of the basic unit is described by eqs. (1)–(3), 2, 3, with the force *F*_*g*_ depending on the degree of swelling *λ*.

We consider the two basic types of electrical connection between the hybrid units: serial (Fig. 2a) and parallel (Fig. 2b). The connected units are assumed to be identical, but can differ in their force polarity. The oscillatory dynamics of each unit is described by eqs. (1)–(3), 2, 3, but the force acting on a given gel now depends on the degrees of swelling of all gels in the system. If *n* units are connected in series, all have the same electric charge, *Q*_*i*_ = *Q*, where *i* = 1,2,…,*n* labels the units, and the net electric potential difference is zero, 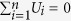. From eqs. (4) and (5), the bending force acting on the *i*^th^ cantilever is:


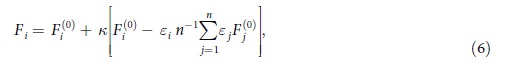


where 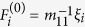 and *κ* = *ζ*^2^(1−*ζ*^2^)^−1^. For the parallel connection, there is no net electric charge in the system, 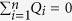, and all bimorphs have the same voltage, *U*_*i*_ = *U*. Hence, the bending force is:


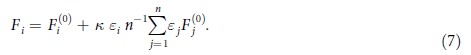


Given eqs. (6) and (7), the forces acting on the *i*^th^ gel can be found as described above, and the latter forces depend on the degrees of swelling of all gels. It is important to note, however, that the cross-terms describing the interaction between the gel-piezoelectric units are small because the interaction is weak, as *κ* is small (see SI).

We first consider two electrically coupled gel-piezoelectric units (Fig. 1a). Numerical solution of the governing equations reveals that the interaction between the units results in synchronization of the chemo-mechanical oscillations of the two BZ gels. Moreover, the mode of synchronization, i.e., the phase difference of oscillations in the synchronized state, depends on the force polarity of the two units; the interacting units exhibit anti-phase synchronization at *ε*_1_ = ε_2_ = 1 (Fig. 3a), or in-phase synchronization if *ε*_1_ = 1 and *ε*_2_ = −1 (Fig. 3b).

The two modes of synchronization could easily be detected through electrical measurements owing to the distinctive patterns of the voltage *U* created by the piezoelectric bimorphs. It can be shown that *U* = −*m*_12_(2*m*_11_*m*_22_)^−1^(1−*ζ*^2^)^−1^(*ξ*_1_ ± *ξ*_2_), where *ξ*_1_ and *ξ*_2_ are the deflections of the two cantilevers, and the (+) and (−) signs in the parentheses correspond to the force polarity sets {1,1} and {1,−1}, respectively (see SI). Hence, the anti-phase gel oscillations at {*ε*_*i*_} = {1,1} generate oscillatory voltage (Fig. 3c), whereas *U* = 0 for the in-phase oscillation at {*ε*_*i*_} = {1,−1} (Fig. 3d). The difference between the waveforms in Fig. 3a,b arises from the different contributions of the piezoelectric effect to the gel-gel interaction for the different sets of *ε*_*i*_ (as detailed in the SI).

The synchronized oscillations in Fig. (3a,b) are the only synchronization modes observed in the simulations at the respective force polarity sets. Figure 3e,f show the evolution of the phase difference Δ*φ* as obtained from 20 independent simulations starting from random initial values of Δ*φ* for the polarity sets {1,1} and {1,−1}, respectively. In all simulations, Δ*φ* converges to *π* in Fig. 3e, and to 0 or 2*π* in Fig. 3f. Figure 3e,f also demonstrate that the process of synchronization is rather slow and might take up to three or four hundred cycles, depending on the initial conditions. The low rate of synchronization between the gel-piezoelectric units is a result of the weak coupling controlled by the small parameter *κ*.

To use these systems for computation, we must first determine the synchronization behavior of *n* electrically connected gel-piezoelectric units. Due to the weak coupling between these units, we can employ the phase dynamics^20^,^21^ approach (see SI), which indicates that the interaction between weakly interacting oscillators only results in the time-dependent deviation of phase in each oscillator, *φ*_*i*_(*t*). For *n* interacting gel-piezoelectric units, the difference of phases *φ*_*ij*_ = *φ*_*i*_−*φ*_*j*_ of oscillators *i* and *j* is:


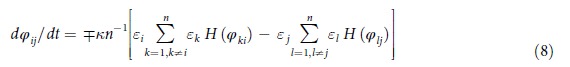


The signs (−) and (+) on the r.h.s. of eq. (8) correspond to the serial and parallel connections of the units, respectively. The connection function *H*(*φ*), which is calculated numerically (see SI), characterizes the rate of phase shift of an individual BZ gel due to the interaction with another gel at the phase difference between the two oscillators of *φ*. Figure 4a shows one cycle of the BZ gel oscillation in an individual gel-piezoelectric unit, and the corresponding connection function is shown in Fig. 4b. The phase normalization is such that 0 ≤ *φ* ≤ 1. Figure 4b shows that interactions can cause positive or negative phase shifts depending on the phase difference.

Using eq. (8), we first demonstrate that the synchronization modes in Fig. 3 are the only modes possible in the system of two gel-piezoelectric units. At *n* = 2, eq. (8) can be written as 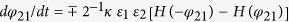. The stable modes of synchronization correspond to those zeroes of the function on the r.h.s. where this function has a negative slope. The function *H*(−*φ*)−*H*(*φ*) (Fig. 4c) has zeroes at *φ* = 0, 0.5, and 1. For the two units connected in parallel (Fig. 1a), *φ*_21_ = 0.5 is the stable solution at *ε*_1_*ε*_2_ > 0 shown in Fig. 3a, and *φ*_21_ = 0 and 1 are the stable solutions at *ε*_1_*ε*_2_ < 0 shown in Fig. 3b.

Equation (8) exhibits useful properties that simplify analysis of synchronization in systems of many gel-piezoelectric units. First, simultaneous change of sign of all the force polarities {*ε*_*i*_} does not change the phase dynamics and thus, does not affect the synchronization modes. Next, the phase dynamics of units connected in series and in parallel differ only in the sign of the function on the r.h.s. of eq. (8). Hence, the steady-states of eq. (8), i.e., the synchronization modes, are the same for the both types of connections. Stability of a steady-state is determined by eigenvalues of the Jacobian matrix, and the eigenvalues have opposite signs for the serial and parallel connections. Therefore, the synchronization modes that are stable for the serial connection are unstable if the units are connected in parallel, and vice versa. Finally, some modes could exhibit the saddle-like stability, and these modes are unstable for both serial and parallel connections of the gel-piezoelectric units.

We use the above properties of eq. (8) to determine all possible modes of synchronization in a system of three gel-piezoelectric units. It is sufficient to consider only two sets of the force polarity of the units, {*ε*_*i*_}, namely, {1,1,1} and {1,1,−1} (For three units, either all the polarities are the same or just one is different.) The respective results are presented in Fig. 5a,b. The synchronization modes are found as the steady-states of two phase difference equations. Specifically, we determine the simultaneous solutions of the equations *dφ*_21_/*dt* = 0 and *dφ*_32_/*dt* = 0. For each of the equations, the solutions form a curve or several curves within the area of the plane with coordinates 0 ≤ *φ*_21_ ≤ 1 and 0 ≤ *φ*_32_ ≤ 1. The intersections of the two sets of curves give the synchronization modes. In Fig. 5a,b, the intersections are marked by colored disks to distinguish between the modes, which are stable for the serial (green) and parallel (red) connections, or are unstable for any of them (grey). Some of the modes are equivalent as they correspond to different combinations of the units or can be obtained by a shift for a period of oscillation.

The insets show the distinct patterns of oscillation for the stable synchronization modes predicted by the phase dynamics equations. There are two distinct modes for both the serial and parallel connections at the force polarity set {1,1,1} (Fig. 5a), and only one mode for each type of connection at the polarities of {1,1,−1} (Fig. 5b). The oscillation patterns were also obtained by solving the full set of equations, eqs. (1)–(3), 2, 3, with the initial conditions corresponding to a chosen synchronization mode perturbed by random phase shift up to 0.01. The latter calculations confirm the results for the stability of the modes obtained from the phase dynamics approach. Having developed this new approach, in future studies, we will investigate large *n* systems.

Due to the synchronization among these oscillators, the system can be utilized to perform such computational tasks as pattern recognition. Our new model allows us to design complex networks of coupled oscillations with arbitrary topologies, as well as predict and control their computational behavior. In its ultimate form, the gel-piezoelectric composite could reside above a porous substrate that contains the reagents for the BZ reaction and the entire system could be encased in a polymeric material. Hence, the system would resemble the gel insets for footwear. Used in this context, pressure from walking could release the BZ reagents and thus, sustain the oscillations. Moreover, the mode of walking would affect the synchronization behavior and thus, the system could detect changes in gait, which could indicate changes in health. More generally, the ability to create smart materials that can sense the environment, process information and react to complex stimuli will enable new systems that will interface with humans to provide tactile, temperature, and photonic inputs to smart clothing.

## Additional Information

**How to cite this article**: Yashin, V. V. *et al.* Achieving synchronization with active hybrid materials: Coupling self-oscillating gels and piezoelectric films. *Sci. Rep.* **5**, 11577; doi: 10.1038/srep11577 (2015).

## Supplementary Material

Supplementary Information

## Figures and Tables

**Figure 1:**
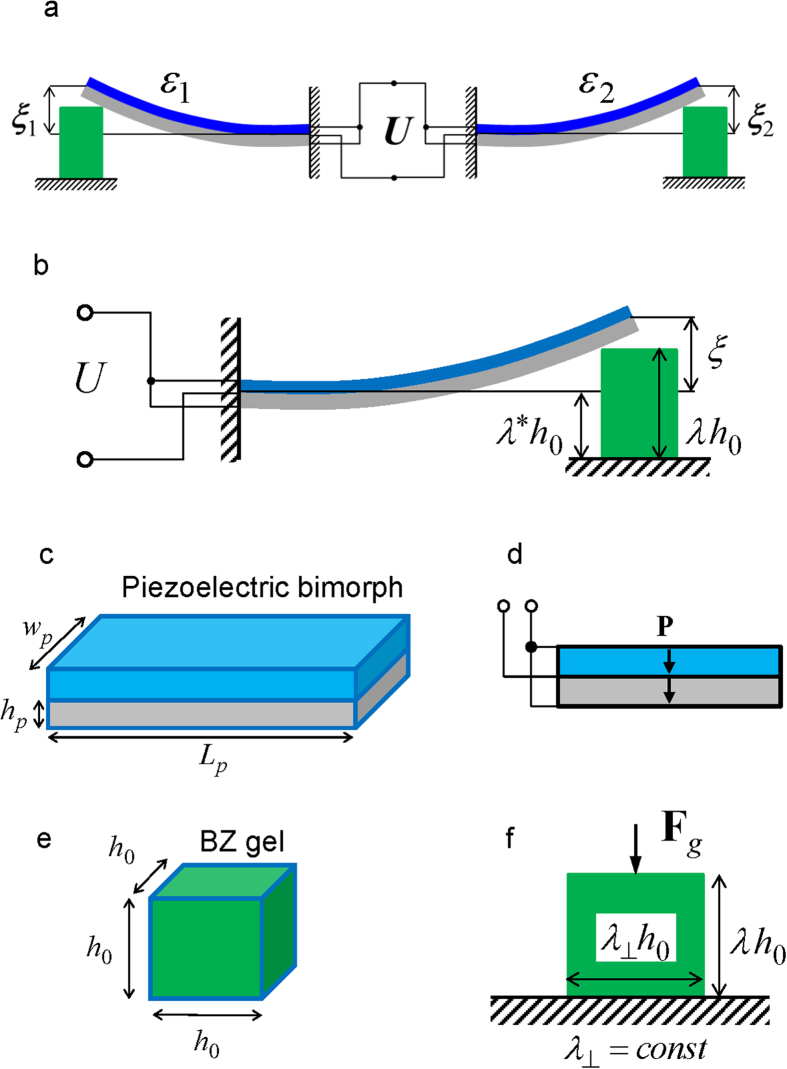
Electrically coupled piezoelectric MEMS actuated by self-oscillating polymer gels. (**a**) Two gel-piezoelectric units coupled through the parallel electric connection. (**b**) The deflection of piezoelectric cantilever *ξ* and the electric potential difference *U* caused by the swollen gel having the degree of swelling *λ*. (**c**) The piezoelectric cantilever consists of two layers having the length *L*_*p*_, width *w*_*p*_, and layer thickness *h*_*p*_. (**d**) The cantilever is fabricated from a polarized piezoelectric material (polarization **P**); the internal and external surface electrodes are connected in parallel. (**e**) An un-deformed gel is cube-shaped of size *h*_0_. (**f**) The gel swelling takes place under the action of force **F**_*g*_, and is restricted to uniaxial deformations characterized by the variable longitudinal and constant transversal degrees of swelling *λ* and *λ*_⊥_, respectively. In calculations, the dimensions are taken to be *h*_0_ = 0.5 mm, *L*_*p*_ = *w*_*p*_ = 1 mm, *h*_*p*_ = 10 μm. The used values λ^*^ and *λ*_⊥_ are discussed in the SI.

**Figure 2:**
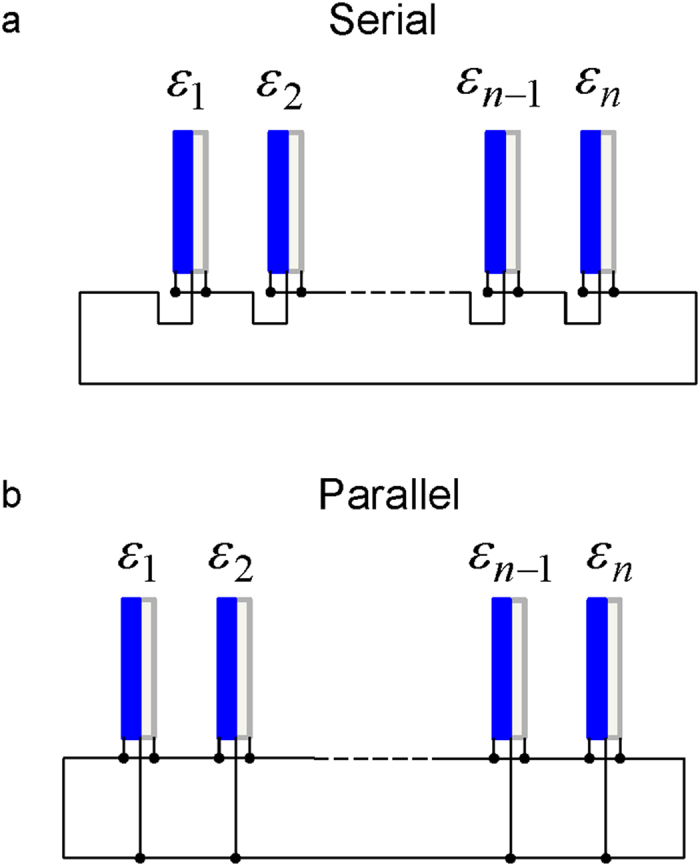
Multiple gel-piezoelectric units connected to circuits. (**a**) Serial connection. (**b**) Parallel connection. The units are identical and can differ only in their force polarity *ε*_*i*_ = ±1, where 1 ≤ *i* ≤ *n* is the unit number.

**Figure 3:**
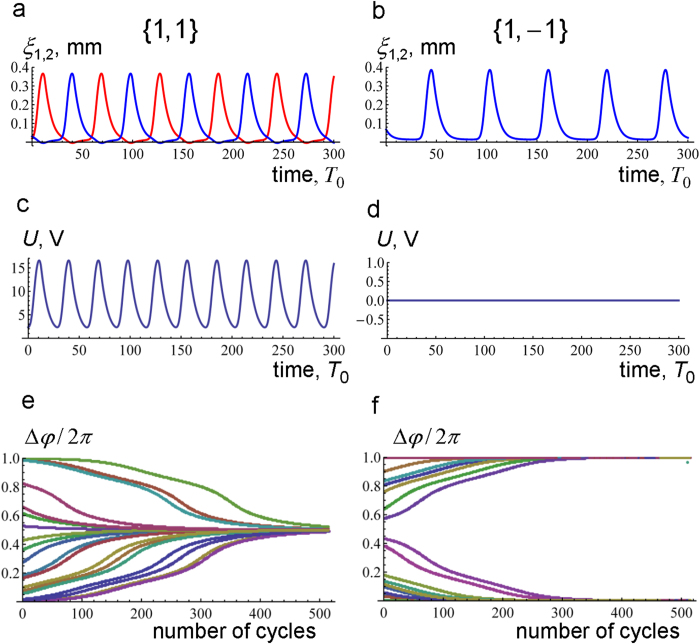
Modes of synchronization in the system of two gel-piezoelectric units. Units are connected in parallel as shown in Fig. 1a. At the force polarity set {*ε*_*i*_} = {1,1}, (**a**) the deflections *ξ*_1_ and *ξ*_2_ oscillate anti-phase, that results in (**c**) the oscillatory voltage *U*. At {*ε*_*i*_} = {1,−1}, (**b**) the cantilevers bend in-phase, and (**d**) no voltage is generated, *U* = 0. No other modes of synchronization are observed in 20 independent simulations starting from random initial values: the phase difference Δ*φ* converges to (**e**) *π* at {*ε*_*i*_} = {1,1}, and to (**f**) 0 or 2*π* at {*ε*_*i*_} = {1,−1}.

**Figure 4:**
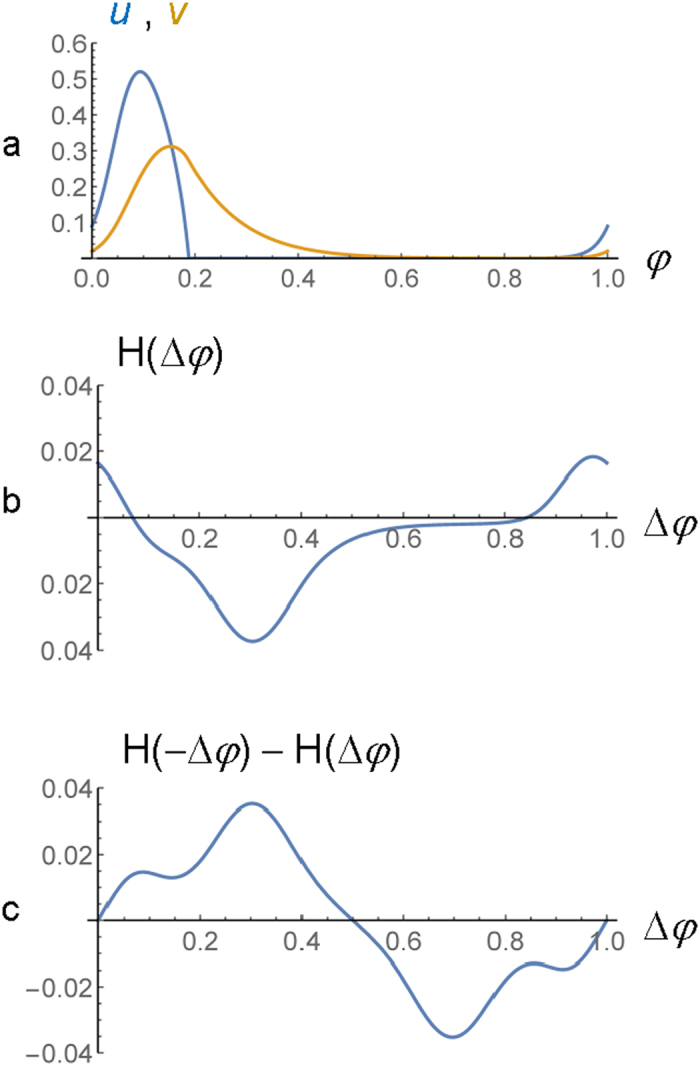
Phase dynamics analysis. (**a**) One cycle of the BZ gel oscillation in an individual gel-piezoelectric unit under no perturbation. (**b**) The connection function *H*(Δ*φ*) that characterizes the rate of phase shift of an individual BZ gel due to the interaction with another gel at the phase difference between the two oscillators of Δ*φ*. (**c**) The function *H*(−Δ*φ*)−*H*(Δ*φ*) describes synchronization in the system of two units.

**Figure 5:**
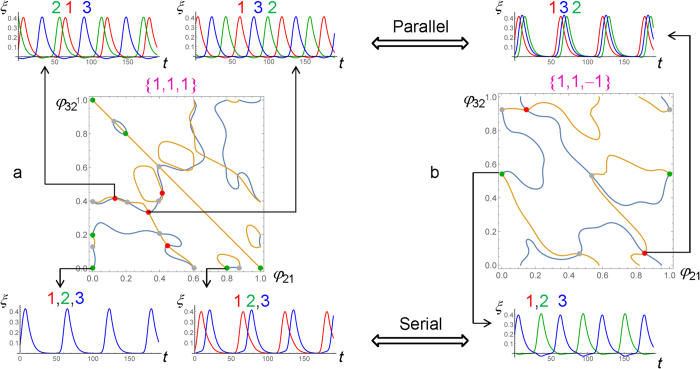
Synchronization modes in systems of three gel-piezoelectric units. Obtained by graphical solution of the equations of phase dynamics for the force polarity sets {*ε*_*i*_} of (**a**) {1,1,1}, and (**b**) {1,1,−1}. The green and red disks mark the modes stable for the serial and parallel connections, respectively. The grey disks mark the modes unstable for the both types of connection. The insets show the cantilever oscillation patterns for the stable synchronization modes. The cantilever deflection curves for the individual units in the system are labeled by the corresponding unit numbers 1, 2, or 3.
