# Supplementary material for: Achieving synchronization with active hybrid materials: Coupling self-oscillating gels and piezoelectric films
Source: Sci Rep. 2015 Jun 24;5:11577. doi: 10.1038/srep11577 (PMC4478467; doi:10.1038/srep11577)
Supplement: Supplementary Information [file srep11577-s1.pdf]

Achieving synchronization with active hybrid materials: Coupling self-oscillating gels and piezoelectric films

Victor V. Yashin[1], Steven P. Levitan[2] and Anna C. Balazs[1]*

[1]Department of Chemical Engineering, [2]Department of Electrical and Computer Engineering, University of Pittsburgh, Pittsburgh, PA 15261, USA

*Corresponding author. E-mail: balazs@pitt.edu

SUPPLEMENTARY INFORMATION

A. List of symbols used in the paper

t	time
u	concentration of activator in the Oregonator model
v	concentration of oxidized catalyst in the Oregonator model
$F_{\mathrm{BZ}}, G_{\mathrm{BZ}}$	reaction rates in the Oregonator model
ϕ	volume fraction of polymer in gel
ϕ_0	volume fraction of polymer in the un-deformed gel
h_0	size of cubic-shaped, un-deformed gel
c_0	gel crosslink density
λ	degree of swelling of gel in the longitudinal direction
$\lambda_{\perp}$	degree of swelling of gel in the transverse direction
λ^*	gel swelling offset at which piezoelectric plate is not deflected
π_{FH}	osmotic pressure of polymer according to the Flory-Huggins theory
χ	polymer-solvent interaction parameter
χ^*	interaction parameter describing hydrating effect of oxidized catalyst
F_g	force acting on gel
ξ	deflection of piezoelectric plate
Q	electric charge of piezoelectric plate
F	force acting on piezoelectric plate
U	voltage applied to piezoelectric plate
m_{11}, m_{12}, m_{22}	coefficients describing electro-mechanic behavior of piezoelectric plate
ε	force polarity of piezoelectric plate
L_p	length of piezoelectric plate
w_p	width of piezoelectric plate
h_p	layer thickness in piezoelectric bimorph plate
E	Young's modulus of piezoelectric material
d_{31}	piezoelectric constant of piezoelectric of material
ε_{33}	dielectric constant of piezoelectric of material

**P**	polarization of piezoelectric material
k	electromechanical coupling factor of piezoelectric material
ζ	dimensionless parameter describing effect of piezoelectricity on bending rigidity
κ	dimensionless parameter describing strength of interaction between units
φ	phase of oscillation
i, j	indexes that label oscillating gel-piezoelectric units
n	number of oscillating units in system
φ_{ij}	difference of phases between oscillating units i and j
H	connection function in phase dynamics equations

B. Kinetics of the BZ reaction in gel

The kinetics of the BZ reaction is described by a modification of the Oregonator model [S1], formulated in terms of the dimensionless concentrations of the key reaction intermediate u ($HBrO_2$, the activator), and the oxidized metal-ion catalyst v (Ru^{3+} in the case considered here). The modified model [S2,S3] accounts for the dependence of the BZ reaction rates on the volume fraction of polymer ϕ, and on the total concentration of catalyst grafted to the network. The reaction rates F_{BZ} and G_{BZ} in eqs. (1) and (2) are determined as follows:

$$F_{\mathrm{BZ}}(u,v,\phi) = (1-\phi)^2 wr - u^2 - (1-\phi) f v \frac{u-q(1-\phi)^2}{u+q(1-\phi)^2} \quad \text{(S1)}$$

$$G_{\mathrm{BZ}}(u,v,\phi) = \varepsilon_{\mathrm{BZ}}[(1-\phi)^2 wr - (1-\phi)v] \quad \text{(S2)}$$

The above reaction rates depend on the dimensionless concentrations of the reduced catalyst Ru^{2+} and the radical BrO_2^*, which are denoted by r and w, respectively [S2]. The concentration r is calculated as:

$$r = c_{Ru}\,\phi_0^{-1}\phi - v\ ,$$

where c_{Ru} and ϕ_0 are the catalyst concentration and volume fraction of polymer in the undeformed gel, respectively. The value of w, the concentration of the radical, is found from the following equation:

$$w = \mu(r^2 + 2u/\mu)^{1/2} - \mu r \ ,$$

where μ is a dimensionless parameter (see ref. 15 for further details). Finally, the stoichiometric factor f and the dimensionless parameter q have the same meaning as in the original Oregonator [S1] model, and we use the notation $\varepsilon_{\mathrm{BZ}}$ for the Oregonator parameter ε [S1].

We assume that the chemical composition of the BZ substrate and the volume fraction of polymer in the undeformed gel are the same as used in the experiments described in ref. [S3], so the corresponding dimensionless parameters are estimated to be $\varepsilon_{\mathrm{BZ}} = 0.212$, $q = 9.52 \times 10^{-5}$, $\mu \approx 7 \times 10^2$, and $\phi_0 = 0.16$. The stoichiometric parameter f and the catalyst content c_{Ru} are the adjustable parameters in the study, and we assume them to be $f = 1$ and $c_{Ru} = 3$.

C. Gel swelling under the action of external force

The stresses acting on the swollen polymer network in the absence of an external force are described by the following general stress-strain equation [S4,S5]:

$$\hat{\boldsymbol{\sigma}} = -\pi_{osm} \hat{\mathbf{I}} + \hat{\boldsymbol{\sigma}}_{\mathrm{el}} \ , \qquad \text{(S3)}$$

where π_{osm} is the osmotic pressure of the polymer, $\hat{\mathbf{I}}$ is the unity tensor, and $\hat{\boldsymbol{\sigma}}_{\mathrm{el}}$ is the contribution to the stress tensor from the elasticity of the cross-linked polymer network. The osmotic pressure of the polymer is calculated as:

$$\pi_{osm} = \pi_{FH}(\phi) + \chi^* v \phi \qquad \text{(S4)}$$

Here, $\pi_{FH} = -[\phi + \log(1-\phi) + \chi(\phi)\phi^2]$ arises from the Flory-Huggins theory, where $\chi(\phi)$ describes the polymer-solvent interactions, and the term $\chi^* v \phi$ describes the hydrating effect of the oxidized catalyst on the responsive gel. The strength of the hydrating effect is controlled by

the coupling parameter $\chi^* > 0$. The gel elasticity contribution to the stress tensor is calculated using the Flory model of rubber elasticity to obtain [S4]:

$$\hat{\boldsymbol{\sigma}}_{\rm el} = -c_0\phi(2\phi_0)^{-1}\hat{\mathbf{I}} + c_0\phi_0^{-1}\phi\hat{\mathbf{B}} \qquad \text{(S5)}$$

Here, c_0 is the cross-link density, ϕ_0 and ϕ are the volume fractions of polymer in the undeformed and deformed gel, respectively, and $\hat{\mathbf{B}}$ is the Finger strain tensor [S6].

The equilibrium degree of swelling of the small gel sample shown in Fig. 1e is determined by balancing all the forces in the gel. In the case of unrestricted swelling in the absence of external forces, the Finger strain tensor in eq. (S5) is $\hat{\mathbf{B}} = \lambda^2\,\hat{\mathbf{I}}$, so the force balance equation, $\hat{\boldsymbol{\sigma}} = 0$, at a given value of v takes the form (see eqs. (S3)-(S5)):

$$c_0\phi_0^{-1}\phi(\lambda^2 - 1/2) = \pi_{FH}(\phi) + \chi^* v\phi \qquad \text{(S6)}$$

Note that for isotropic swelling, the volume fraction of gel ϕ and the degree of swelling λ are related as $\phi = \phi_0\,\lambda^{-3}$. Hence, solving eq. (S6) yields the degree of swelling λ as a function of the concentration of oxidized catalyst, v.

If the external force acts on the gel as shown in Fig. 1f, the gel deformation is described by the strain tensor

$$\hat{\mathbf{B}} = \begin{pmatrix} \lambda^2 & 0 & 0 \\ 0 & \lambda_\perp^2 & 0 \\ 0 & 0 & \lambda_\perp^2 \end{pmatrix},$$

where λ and $\lambda_\perp$ are the degrees of swelling in the longitudinal and transverse directions, respectively. The equilibrium degree of swelling is determined by balancing all the forces in the two directions, namely,

$$c_0\phi_0^{-1}\phi(\lambda^2 - 1/2) + (h_0\lambda_\perp)^{-2}\,F_g = \pi_{osm} \qquad \text{(S7)}$$

$$c_0\phi_0^{-1}\phi(\lambda_\perp^2 - 1/2) = \pi_{osm} \quad \text{(S8)}$$

Here, h_0 is the undeformed gel size (see Fig. 1e), the osmotic pressure π_{osm} is calculated according to eq. (S4), and the volume fraction of polymer depends on the degrees of swelling λ and $\lambda_\perp$ as $\phi = \phi_0 \lambda^{-1}\lambda_\perp^{-2}$. Therefore, eqs. (S7) and (S8) determine the values of λ and $\lambda_\perp$ as functions of the concentration of oxidized catalyst v and the force F_g.

Upon confinement in a capillary, $\lambda_\perp = const$ and eq. (S7) provides the degree of swelling λ as a function of v, and F_g. (Equation (3) in the paper is eq. (S7) written in a slightly different form.)

In the paper, the volume fraction of polymer and the crosslink density in the undeformed BZ gel used to obtain Fig. 3 are $\phi_0 = 0.139$ and $c_0 = 4.5\times10^{-4}$, respectively, and those used to obtain Fig. 4 and 5 are $\phi_0 = 0.16$ and $c_0 = 4\times10^{-4}$. The polymer-solvent interactions are characterized by the function $\chi(\phi) = 0.338 + 0.518\phi$, which is known to describe the PNIPAAm-water interaction at 20°C [S7]. The interaction parameter χ^*, which accounts for the hydrating effect of the oxidized catalyst, is an adjustable parameter of the model; we set $\chi^* = 0.105$ in this study. The same value of χ^* was used in our previous publications [S2,S4]. Finally, the undeformed gel size is $h_0 = 0.5\,\text{mm}$.

The values of $\lambda_\perp$ and of the offset λ^* (see Fig. 1b) used to obtain Fig 3 are $\lambda_\perp = 1.4$ and $\lambda^* = 1.55$. In Fig. 4 and 5, the values of $\lambda_\perp$ and λ^* are taken equal to λ_{st}, which corresponds to the steady-state value for an isotropic BZ gel. To calculate λ_{st}, the equation for isotropic swelling, eq. (S6), is solved simultaneously with the equations for the steady-state of the BZ

reaction $F_{\mathrm{BZ}}(u,v,\phi)=0$ and $G_{\mathrm{BZ}}(u,v,\phi)=0$ (see eqs. (S1) and (S2)) at $\phi=\phi_0\,\lambda^{-3}$. At the values of model parameters given above, $\lambda_{\perp}=\lambda^{*}=\lambda_{st}\approx 1.65$.

D. Properties of a bending piezoelectric bimorph plate

The coefficients m_{11}, m_{12}, and m_{22} in eqs. (4) and (5) that describe the behavior of a bending piezoelectric bimorph plate are given by the following equations [S8]:

$$m_{11}=\frac{(L_p/h_p)^3}{2w_pE} \tag{S9}$$

$$m_{12}=\frac{3}{4}d_{31}(L_p/h_p)^2 \tag{S10}$$

$$m_{22}=2\,w_p\varepsilon_{33}(1-k^2/4)(L_p/h_p) \tag{S11}$$

Here, L_p, w_p, and h_p are the respective length, width, and layer thickness of the piezoelectric bimorph plate (see Fig. 1c); E, d_{31}, and ε_{33} are the Young's modulus, piezoelectric constant, and dielectric constant of the piezoelectric material, respectively. Finally, $k=(d_{31}^2E/\varepsilon_{33})^{1/2}$ is the electromechanical coupling factor characterizing the piezoelectric material. The piezoelectric bimorph plate dimensions are taken to be $L_p=w_p=1\,\mathrm{mm}$, $h_p=10\,\mu\mathrm{m}$. The plate is assumed to be fabricated from polarized Lead-Zirconate-Titanate (PZT) ceramics. A typical PZT ceramic has a Young's modulus of $E=50\,\mathrm{GPa}$; typical values for the other parameters are $d_{31}=-1.5\times10^{-10}\mathrm{mV}^{-1}$ and $\varepsilon_{33}=1.8\times10^{3}\varepsilon_0$, where $\varepsilon_0=8.85\,\mathrm{pFV}^{-1}$ is the dielectric constant of vacuum [S9]. In our calculations, we use the latter values of E and ε_{33}, and a twofold greater value for the piezoelectric constant (setting $d_{31}=-3\times10^{-10}\mathrm{mV}^{-1}$), which can be achieved in PZT through sophisticated processing methods [S10,S11].

It follows from eqs. (S9)-(S11) that $\zeta^2 = m_{12}^2 (m_{11} m_{22})^{-1} = 9k^2 (16 - 4k^2)^{-1}$ does not depend on the bimorph dimensions. The strength of the interaction between electrically coupled gel-piezoelectric units is controlled by the parameter $\kappa = \zeta^2 (1 - \zeta^2)^{-1}$ (see eqs. (6) and (7) and below). At the model parameter values used for calculations, we obtain $k \approx 0.531$, $\zeta \approx 0.413$, and $\kappa \approx 0.206$. For comparison, the latter dimensionless values at $d_{31} = -1.5 \times 10^{-10} \mathrm{mV}^{-1}$(i.e., for PZT ceramics obtained under common processing conditions) are $k \approx 0.268$, $\zeta \approx 0.201$, and $\kappa \approx 0.042$.

E. Electrically connected gel-piezoelectric units

To obtain eqs. (6) and (7), we start with eqs. (4) and (5) for a system of n units, where $i = 1,2,\ldots,n$ labels the units:

$$\xi_i = m_{11} F_i + m_{12} \varepsilon_i U_i$$

$$Q_i = m_{12} \varepsilon_i F_i + m_{22} U_i$$

(In this section, Q represents change; $Q(\theta)$ has a distinct meaning, representing the phase response curve (PRC) as defined in the section below.) It is convenient to re-write these equations as:

$$F_i = F_i^{(0)} - m_{11}^{-1} m_{12} \varepsilon_i U_i \tag{S12}$$

$$Q_i = m_{12} \varepsilon_i F_i^{(0)} + m_{22} (1 - \zeta^2) U_i \tag{S13}$$

Here, $F_i^{(0)} = m_{11}^{-1} \xi_i$ is Hook's law for an individual bending plate without accounting for the piezoelectric effect (see eq. (S9) for m_{11}), and $\zeta^2 = m_{12}^2 (m_{11} m_{22})^{-1}$. For the serial connection

(see Fig. 2a), $Q_i = Q$ and $\sum_{i=1}^{n} U_i = 0$. Summation of equations (S13) for all $1 \le i \le n$ gives $Q = m_{12} n^{-1} \sum_{j=1}^{n} \varepsilon_j F_j^{(0)}$. Therefore,

$$U_i = -m_{12} m_{22}^{-1} (1-\zeta^2)^{-1} [\varepsilon_i F_i^{(0)} - n^{-1} \sum_{j=1}^{n} \varepsilon_j F_j^{(0)}] \tag{S14}$$

and, after substituting eq. (S14) into eq. (S12),

$$F_i = F_i^{(0)} + \kappa [F_i^{(0)} - \varepsilon_i n^{-1} \sum_{j=1}^{n} \varepsilon_j F_j^{(0)}] \ , \tag{S15}$$

where $\kappa = \zeta^2 (1-\zeta^2)^{-1}$.

For the parallel connection (see Fig. 2b), $U_i = U$ and $\sum_{i=1}^{n} Q_i = 0$. Summation of eq. (S13) yields

$$U = -m_{12} m_{22}^{-1} (1-\zeta^2)^{-1} n^{-1} \sum_{j=1}^{n} \varepsilon_j F_j^{(0)} \tag{S16}$$

and

$$F_i = F_i^{(0)} + \kappa \varepsilon_i n^{-1} \sum_{j=1}^{n} \varepsilon_j F_j^{(0)} \tag{S17}$$

Equations (S15) and (S17) yield eqs. (6) and (7), respectively.

We now discuss how the piezoelectric coupling affects the waveforms in Figs. 3a and 3b. For this purpose, we use eqs. (S16) and (S17) to obtain the equation for the deflections ξ_i ($i = 1,2,\ldots,n$) of the cantilevers, which are connected in parallel and experience forces F_j ($j = 1,2,\ldots,n$). In particular, we find that: $\xi_i = m_{11} F_i - m_{11} \zeta^2 \varepsilon_i n^{-1} \sum_{j=1}^{n} \varepsilon_j F_j$. Each force is

assumed to be positive and vary from approximately zero to some maximal value. In the synchronized state, the forces have the same wave form, and are shifted in phase relative to each other. In the case of $n = 2$ shown in Fig. 3, the deflection of cantilever 1 is calculated as $\xi_1 = m_{11}[F_1 - \zeta^2 (F_1 \pm F_2)/2]$. Here, the $(+)$ and $(-)$ signs in the parentheses correspond to the respective force polarity sets $\{\varepsilon_i\} = \{1,1\}$ and $\{1,-1\}$. For $\{\varepsilon_i\} = \{1,1\}$, the system displays anti-phase synchronization (Fig. 3a). Correspondingly, the force F_2 exhibits a maximum when the force F_1 is at the minimal value, $F_1 \approx 0$, and hence, the deflection of cantilever 1 exhibits a "dip" $\xi_1 \approx -m_{11}\zeta^2 F_2 / 2$ at these moments of time, as is clearly seen in Fig. 3a. These dips are relatively shallow because of the small value of the parameter ζ^2, which characterizes the strength of the piezoelectric effect on the bending of the plate. If $\{\varepsilon_i\} = \{1,-1\}$, the units are synchronized in-phase, so that $F_1 = F_2 = F$ and $\xi_1 = \xi_2 = m_{11}F$, i.e., piezoelectricity does not contribute to the cantilever deflections, which follow the applied force and do not exhibit any dips, as seen in Fig. 3b. Note that similar arguments apply to the waveforms shown in Fig. 5 for the case of $n = 3$.

F. Equations of phase dynamics

The phase dynamics approach is an approximation used for describing the behavior of interacting oscillators in the limit of weak coupling [S12,S13]. The system of n identical oscillators is assumed to be governed by the following set of equations

$$dx_i/dt = f(x_i) + \mu \sum_{j=1}^{n} g_{ij}(x_i, x_j) \ , \qquad \text{(S18)}$$

where x_i is the set of variables describing the i-th oscillator, $g_{ij}(x_i, x_j)$ determines the interaction between oscillators i and j, and the parameter μ controls the strength of the interaction. In the case of no interaction ($\mu = 0$), all oscillators exhibit the same T-periodic stable limit cycle behavior $\bar{x}(t+T) = \bar{x}(t)$. If the coupling is weak ($\mu << 1$), the interaction results primarily in a deviation of the phase of oscillation, and the solution of eq. (S18) takes the form $x_i(t) = \bar{x}(t+\varphi_i)$, where φ_i is the oscillation phase.

The evolution of the phase in time is described by the following equation [S13]:

$$d\varphi_i/dt = \mu Q(t+\varphi_i)\sum_{j=1}^{n} g_{ij}(\bar{x}(t+\varphi_i), \bar{x}(t+\varphi_j)) \qquad \text{(S19)}$$

The T-periodic function (vector function) $Q(\theta)$ is known as the phase response curve (PRC) [S13]; it is also called the phase projection vector (PPV) [S14,S15]. The PRC can be determined using Malkin's method [S12,S13]. Namely, the function $Q(\theta)$ is the solution of the "adjoint" equation

$$dQ/dt = -\{Df(\bar{x}(t))\}^{\mathrm{T}} Q \ , \qquad \text{(S20)}$$

under the initial condition $Q(0) \bullet f(\bar{x}(0)) = 1$. In eq. (S20), $\{Df(\bar{x}(t))\}$ is the Jacobian matrix for a free-running oscillator (see (S18)) calculated along the limit cycle.

Solutions of eq. (S19) exhibit both the fast dynamics on the time scale of the period of oscillation T and the slow dynamics on the time scale of μ^{-1}. Note that the synchronization phenomena take place on the slow time scale. To extract the slow dynamics, time averaging is applied to eq. (S19) to obtain [S12]:

$$d\varphi_i/dt = \mu \mathrm{H}_{ii}(0) + \mu \sum_{j \neq i}^{n} \mathrm{H}_{ij}(\varphi_j - \varphi_i) \ , \qquad \text{(S21)}$$

where

$$\mathrm{H}_{ij}(\varphi_j - \varphi_i) = T^{-1}\int_0^T Q(t)\cdot g_{ij}(\bar{x}(t), \bar{x}(t+\varphi_j - \varphi_i))\,dt \tag{S22}$$

To apply the above formalism to the gel-piezoelectric units, we introduce the partial variables for the BZ reactants $\mathsf{u} = u(1-\phi)^{-1}$ and $\mathsf{v} = v\phi^{-1}$, and write eqs. (1) and (2) for a free-running unit as

$$d\mathsf{u}/dt = \mathsf{F}(\mathsf{u},\mathsf{v},\phi(\mathsf{v})) \tag{S23}$$

$$d\mathsf{v}/dt = \mathsf{G}(\mathsf{u},\mathsf{v},\phi(\mathsf{v})) \tag{S24}$$

where $\mathsf{F} = (1-\phi)^{-1}F_{\mathrm{BZ}}$ and $\mathsf{G} = \phi^{-1}G_{\mathrm{BZ}}$. In eqs. (S23) and (S24), $\phi(\mathsf{v})$ is the solution of the equilibrium swelling equation (see eqs. (3) and (S7)), conveniently written in the following form:

$$\pi_{\mathrm{FH}}(\phi) + \chi^{*}\mathsf{v}\phi^{2} - \sigma_{el}(\phi) - F(\phi) = 0\ . \tag{S25}$$

Here $\sigma_{el}(\phi)$ and $F(\phi)$ are the respective elastic stress in the gel and the pressure on the gel due to the elastic bending plate, expressed as functions of the volume fraction of polymer, ϕ. In the case of uniaxial deformation of the gel, $\sigma_{el}(\phi) = c_0[\lambda(\phi)\lambda_{\perp}^{-2} - \phi(2\phi_0)^{-1}]$, where $\lambda(\phi) = \phi_0\lambda_{\perp}^{-2}\phi^{-1}$, and $F(\phi) = (h_0\lambda_{\perp}^{2}m_{11})^{-1}[\lambda(\phi) - \lambda^{*}]$.

When the gel-piezoelectric units are electrically connected, the force exerted on a gel is $F + \delta F$, where δF is the perturbation resulting from the interaction due to the piezoelectric effect (here, for simplicity, we dropped the subscript index labeling units). On the right-hand side of eqs. (6) and (7), δF is given by the second terms, and the factor κ controls the interaction strength. To represent the effect of interaction in the form of eq. (S18), eqs. (S23) and (S24) are expanded into a Taylor series to obtain:

$$d\mathsf{u}/dt = \mathsf{F}(\mathsf{u},\mathsf{v},\phi(\mathsf{v})) + \mathsf{F}_{\phi}(\mathsf{u},\mathsf{v},\phi(\mathsf{v}))\,K^{-1}(\mathsf{v})\,\delta F \tag{S26}$$

$$d\mathsf{v}/dt = \mathsf{G}(\mathsf{u},\mathsf{v},\phi(\mathsf{v})) + \mathsf{G}_\phi(\mathsf{u},\mathsf{v},\phi(\mathsf{v}))\, K^{-1}(\mathsf{v})\,\delta F \quad , \tag{S27}$$

where the subscript "ϕ" denotes the partial derivative $\partial/\partial\phi$, and

$$K(\mathsf{v}) = \partial_\phi[\pi_{FH}(\phi) + \chi^* \mathsf{v}\phi^2 - \sigma_{el}(\phi) - F(\phi)]\Big|_{\phi=\phi(\mathsf{v})} \tag{S28}$$

The explicit forms of eq. (S18) and hence the function g_{ij} are obtained from eqs. (S26) and (S27) after taking into account eqs. (6) and (7) that describe interaction between the units connected in series and in parallel, respectively. For example, for the parallel connection, we obtain the following equation:

$$\frac{d}{dt}\begin{pmatrix}\mathsf{u}\\ \mathsf{v}\end{pmatrix}_i = \begin{pmatrix}\mathsf{F}\\ \mathsf{G}\end{pmatrix}_i + \kappa\sum_{j=1}^{n} n^{-1}\varepsilon_i\varepsilon_j \begin{pmatrix}\mathsf{F}_\phi K^{-1}\\ \mathsf{G}_\phi K^{-1}\end{pmatrix}_i F_j^{(0)} \quad . \tag{S29}$$

On the r.h.s. of eq. (S29), the column-vectors having the subscript "i" depend on u and v in the unit i, and $F_j^{(0)}$ depends on the volume fraction of polymer (see the paper) and thus, on v in the unit j. Note that in eq. (S29), the parameter κ plays the role of the interaction parameter μ in eq. (S18).

The limit cycle solution of eqs. (S23) and (S24) shown in Fig. 4a is obtained numerically and then used to solve eq. (S20) to determine the PRC functions Q_u and Q_v. The Jacobian matrix $\{Df(\bar{x}(t))\}$ is calculated as

$$D\{f(\bar{x})\} = \begin{pmatrix}\mathsf{F}_\mathsf{u} & \mathsf{F}_\mathsf{v} - \chi^*\phi^2 K^{-1}\mathsf{F}_\phi\\ \mathsf{G}_\mathsf{u} & \mathsf{G}_\mathsf{v} - \chi^*\phi^2 K^{-1}\mathsf{G}_\phi\end{pmatrix}$$

where K is defined by eq. (S28), and the subscripts denote the partial derivative with respect to the corresponding variable. Numerical integration of eq. (S20) is performed backwards as

described in ref. [S13]. Finally, the connection function $H(\Delta\varphi)$ shown in Fig. 4b is obtained through the time averaging procedure, eq. (S22).

References

S1. J. J. Tyson and P. C. Fife, “Target patterns in a realistic model of the Belousov–Zhabotinskii reaction”, *J. Chem. Phys.*, 1980, **73**, 2224–2237.

S2. V. V. Yashin, O. Kuksenok and A. C. Balazs, “Computational design of active, self-reinforcing gels”, *J. Phys. Chem. B*, 2010, **114**, 6316–6322.

S3. V. V Yashin, S. Suzuki, R, Yoshida and A. C. Balazs, “Controlling the dynamic behavior of heterogeneous self-oscillating gels”, *J. Mater. Chem.*, 2012, **22**, 13625-13636.

S4. V. V. Yashin and A. C. Balazs, “Theoretical and computational modeling of self-oscillating polymer gels”, *J. Chem. Phys.*, 2007, **126**, 124707.1-124707.17.

S5. V. V. Yashin and A. C. Balazs, “Modeling polymer gels exhibiting self-oscillations due to the Belousov-Zhabotinsky reaction”, *Macromolecules*, 2006, **39**, 2024–2026.

S6. R. J. Atkin and N. Fox, *An Introduction to the Theory of Elasticity*, Longman, New York, 1980.

S7. S. Hirotsu, “Softening of bulk modulus and negative Poisson’s ratio near the volume phase transition of polymer gels”, *J. Chem. Phys.*, 1991, **94**, 3949–3957.

S8. R. G. Ballas, *Piezoelectric Multilayer Beam Bending Actuators: Static and Dynamic Behavior and Aspects of Sensor Integration*, Springer, New York, 2007.

S9. A. Preumont, *Mechatronics : Dynamics of Electromechanical and Piezoelectric Systems*, Springer, Dortrecht, 2006.

S10. S. H. Baek, J. Park, D. M. Kim, *et al.*, “Giant piezoelectricity on Si for hyperactive MEMS”, *Science*, 2011, **334**, 958-961.

S11. S. H. Baek, M. S. Rzchowski and V. A. Aksyuk, “Giant piezoelectricity in PMN-PT thin films: Beyond PZT”, *MRS Bulletin*, 2012, **37**, 1022-1029.

S12. F. C. Hoppensteadt and E. M. Izhikevich, *Weakly Connected Neural Networks*, Springer, New York, 2007.

S13. E. M. Izhikevich, *Dynamical Systems in Neuroscience: The Geometry of Excitability and Bursting*, MIT Press, Cambridge, 2007, Chapter 10.

S14. A. Demir, A. Mehrotra and J. Roychowdhury, “Phase noise in oscillators: a unifying theory and numerical methods for characterization”, *IEEE Trans. on Circuits and Systems – I: Fundamental Theory and Applications*, 2000, **47**, 655-674.

S15. X. Lai and J. Roychowdhury, “Fast simulation of large networks of nanotechnological and biochemical oscillators for investigating self-organization phenomena”, *Asia and South Pacific Conference on Design Automation (Yokohama, 2006)*, DOI: 10.1109/ASPDAC.2006.1594694.
